# Quantum Computing for Transport Network Optimization

**DOI:** 10.3390/e27090953

**Published:** 2025-09-13

**Authors:** Jiangwei Ju, Zhihang Liu, Yuelin Bai, Yong Wang, Qi Gao, Yin Ma, Chao Zheng, Kai Wen

**Affiliations:** 1 Beijing QBoson Quantum Technology Co., Ltd., Beijing 100015, China; 2 School of Energy Storage Science and Engineering, North China University of Technology, Beijing 100144, China

**Keywords:** coherent Ising machine, quantum computing, bus route optimization, QUBO model

## Abstract

Public transport systems play a crucial role in the development of large cities. Bus network design to optimize passenger flow coverage in a global metropolis is a challenging task. As an essential part of bus travel planning, considering the bus transfer factor in the existing extremely complex and extensive public bus network usually leads to a optimization problem characterized by high-dimensionality and non-linearity. While classical computers struggle to deal with this kind of problems, quantum computers shed new light into this field. The coherent Ising machine (CIM), a specialized optical quantum computer using a photonic dissipative architecture, has shown its remarkable computational power in combinatorial optimization problems. We construct the classical model and the quadratic unconstrained binary optimization (QUBO) model of the bus route optimization problem, and solve it using a classical computer and CIM, respectively. Our experimental results demonstrate the significant acceleration capability of CIM over classical computers in finding the optimal or near-optimal solutions, albeit subject to the hardware limitations of the 100-qubit CIM.

## 1. Introduction

For the last several decades, quantum computing has been showing its beyond-classical computation capability for certain problems [1,2,3,4,5,6]. In recent years, with the gradual maturation of quantum computing technology, the application of practical quantum computers to real-world problems has been attracting increasing attention. In other words, the development of quantum computing is no longer confined to its scientific significance, as its social and industrial value is gradually becoming evident and substantiated [7].

In particular, the rapid progress in quantum computing has unlocked unprecedented potential for tackling complex challenges in diverse fields such as transport network design (TND). As a multidisciplinary domain, TND aims at enhancing the efficiency and sustainability of transportation networks by integrating numerous technologies and applications [7,8]. Nevertheless, practical TND is usually an NP-hard problem as we need to process enormous datasets and solve intricate optimization problems associated with it. While classical computational methods struggle to deal with it, quantum computing, with its distinctive advantages in parallel computation, optimization algorithms based on the principle of quantum mechanics, and machine learning applications, presents transformative opportunities for revolutionizing transportation system management and optimization [7,8,9,10]. By way of example, the bus network is an indispensable fundamental component of transportation infrastructure, yet multi-objective large-scale bus route optimization (BRO) problem is computationally challenging for classical computers. More specifically, bus network design is a crucial problem in urban transportation planning and the corresponding optimization problem involves making different decisions such as station selection and operation scheduling. The first bus network design framework was systematically proposed by Ceder and Wilson, which divides the problem into three levels: route design, frequency optimization, and scheduling [11]. In general, the objectives of bus network optimization include minimizing operating costs, maximizing service coverage and improving passenger satisfaction. Adding a new bus route by choosing from a set of candidate stations is a real-world problem, which could lead to better service coverage and passenger satisfaction. Focusing on optimizing route length and station spacing, a genetic algorithm-based bus route optimization method was proposed [12]. As passenger flow data becomes easier to access, demand-based optimization goals are attracting more and more attention. For example, bus route design should prioritize coverage of high-demand areas, which leads to the introduction of passenger flow coverage as an optimization metric [13]. To improve the connectivity and coverage of the bus network, bus transfer, an important feature of the bus transportation system, has been incorporated into the bus route planning model [14,15,16,17]. While much progress has been made in the field of bus network optimization, adding a new bus route by choosing from a set of candidate stations to maximize passenger flow coverage is a large-scale optimization problem and presents many challenges. Classical computers always struggle to solve the problem efficiently when the number of candidate stations is large and bus transfer needs to be considered, as we usually encounter in the bus network design of metropolis.

The coherent Ising machine (CIM), an optical-system-based specialized quantum computer designed for solving combinatorial optimization problems and applicable across various fields [18,19,20,21,22,23,24,25,26,27], is well suited for addressing multi-objective large-scale BRO problems. Most combinatorial optimization problems can be modeled as quadratic unconstrained binary optimization (QUBO) problems, and the QUBO model is equivalent to the Ising model in physics, which can be effectively simulated by CIM. By mapping optimization problems to the ground state search problems of the corresponding Ising models, CIM can efficiently search for the global optimal solution utilizing the coherence and nonlinear characteristics of the Optical Parametric Oscillator (OPO) [18,21,22], which is the core component of CIM. In particular, networks of OPOs have been proven to be able to generate spatial multimode entanglement [20]. Compared with classical computers and quantum annealers, CIM features high parallelism and low energy consumption. Moreover, CIM is robust to environment noise and can operate at room temperature environment, making its practical application more accessible than quantum computers based on superconducting circuits.

In this paper, we construct the QUBO model for BRO problems and solve it using CIM for the first time. To compare the computation efficiency of CIM and classical computers, the classical model and the QUBO model of the bus route optimization problem are constructed and solved using a classical computer and CIM, respectively. Our experimental results show the significant acceleration capability of CIM, albeit subject to the hardware limitations of the 100-qubit CIM. We expect that the advantage of CIM in solving large-scale optimization problems will grow rapidly along with the development of hardware capabilities of coherent Ising machine with thousands of qubits.

## 2. Bus Route Optimization Model

We aim to design a new route from the set of candidate stations *S* to maximize passenger flow coverage, a critical metric directly reflecting the route’s ability to meet potential passenger demand. The main challenge of this problem stems from that we must consider bus transfer in the complex and extensive bus network of metropolis. Bus transfer is an essential part of bus travel planning, and the connection with other bus routes can significantly expand the coverage, which also significantly increases the complexity and computational difficulty of the BRO model [15,16,17], especially when we are considering a global metropolis such as Beijing. This problem is naturally related to multi-objective optimization, graph theory and algorithm design, showing both theoretical and practical significance [13,14,28]. For example, BRO often needs to balance multiple competing objectives, such as maximizing service coverage to ensure passenger accessibility, minimizing route directness to reduce travel time, and optimizing operational costs for efficiency [13]. The bus network can be modeled using graph theory, with the bus station as the node and the route as the edge, and then the shortest path algorithm can be applied to design the efficient route [11,13].

### 2.1. Classical Model of Bus Route Optimization

The real-world bus route optimization problem we are going to address involves at least 60 candidate stations in Beijing, and as we shall take bus transfer into consideration, the extremely complex and extensive public bus network of Beijing makes the problem difficult to tackle. Indeed, due to the complex solution spaces, multi-local extremums, and computational complexity, high-dimensional, non-convex, or large-scale combinatorial optimization problems are challenging for traditional optimization methods. For certain cases, we can adopt the two-stage optimization strategy to effectively address these problems [29,30]. By decomposing the problem into two phases, pre-optimization phase and refining phase, we first find the potential high-quality solution subspace and then search for global optimal solution in the refining phase. To improve clarity, we present the pseudocode Algorithm A1 in Appendix A to illustrate the two-stage pipeline. Considering the complexity of the problem and the hardware limitations of the 100-qubit CIM we used, we adopt a similar strategy to solve the BRO problem. In the pre-optimization phase, OR-Tools [31] is used to generate a high-quality initial guess. Specifically, the CP-SAT solver with default configuration is used. For the entire project, the programming environment is Python 3.10.

In the pre-optimization phase, the objective function and constraints of the classical model are described below. The mathematical notation of the problem is explained in Table 1. When bus transfer is not taken into consideration explicitly, the following general constraints are imposed. Stations are denoted as *i*, *j*, or *k* and we denote *E* the set of all stations and S⊆E the set of candidate stations for the new bus route. Origin–destination (OD) pairs are denoted as (i,j) and the set of OD pairs is W⊆E×E. With *A* being the set of adjacent stations, an OD pair (i,j)∈A if and only if the distance $d_{i,j}$ between stations *i* and *j* does not exceed a given threshold $L_{a}$. We then construct the the adjacency matrix $\left(a_{ij}\right)$, with $a_{ij}=1$ if and only if (i,j)∈A and otherwise $a_{ij}=0$. We set $x_{ij}$ the binary variables indicating whether the path from station *i* to station *j* is selected in the new bus route, with (i,j)∈A. Thus, we have

(1) $$x_{ij}\leq a_{ij},\;i,j\in E,$$

which ensures that arcs can only be used if (i,j)∈A. For the start station *s* of the new bus route,

(2) $$\sum_{j\in S}x_{sj}=1$$

ensures that exactly one arc leaves the start station *s*; for the terminal station *t*,

(3) $$\sum_{i\in S}x_{it}=1$$

ensures that exactly one arc enters the terminal station *t*; and for all intermediate stations i∈S∖{s,t},

(4) $$\sum_{j\in S}x_{ij}=\sum_{k\in S}x_{ki},\;\forall i\in S\setminus \left\{s,t\right\}$$

ensures flow conservation at intermediate stations. The total length of the bus route is related to the city’s scale and the average travel distance of its residents. For urban public bus network, the length of the main routes should ideally be 8–12 km, while for small and medium-sized cities, the lower limit can be relaxed appropriately, and for distinctly linear cities, the upper limit can be relaxed. Overly long bus routes may lead to uneven passenger flow distribution, affecting operational efficiency, while overly short routes may increase passenger transfers and waiting times, negatively impacting the overall scheduling of the bus network. Setting $L_{\min}$ and $L_{\max}$ as the lower and upper bound of the total length of the new bus route respectively, we have

(5) $$L_{\min}\leq \sum_{i,j\in S}d_{ij}\cdot x_{ij}\leq L_{\max}.$$

The non-linearity coefficient of a bus route is the ratio of the actual bus route distance to the straight-line distance $L_{s}$ between the given start station *s* and terminal station *t* and is upper bounded by $L_{\max}/L_{s}$. A smaller coefficient is preferable, and usually, the non-linearity coefficient for a single route should not exceed 1.4. Set a binary variable $\delta_{i}$ indicating whether the new bus route passes through station i∈S. $\delta_{i}=1$ if yes, otherwise $\delta_{i}=0$.

(6) $$\delta_{i}\leq \sum_{k\in S}x_{ik}.$$

If the new bus route passes through both stations *i* and *j*, the travel demand between them is satisfied. Thus, $\delta_{i}\cdot \delta_{j}$ indicates whether the new route can meet the travel demand from station *i* to station *j*. With consideration of the coverage of existing bus network, we define $\theta_{ij}=1$ if station i∈S can reach station j∈E through existing routes, otherwise $\theta_{ij}=0$. By definition, $\theta_{ii}=1$. Taking bus transfer into consideration explicitly, we define the following: Binary variable $\Delta_{ij}^{\left(1\right)}=1$ indicates that stations *i* and *j* are directly connected, i.e., no transfer is needed; otherwise, $\Delta_{ij}^{\left(1\right)}=0$; binary variable $\Delta_{ij}^{\left(2\right)}=1$ indicates that stations *i* and *j* are connected via transfers; otherwise, $\Delta_{ij}^{\left(2\right)}=0$. We then have the following constraints:

(7) $$\Delta_{ij}^{\left(2\right)}\leq \delta_{i}+\delta_{j},\;i,j\in E,$$

constraint (7) ensures that if the new bus route does not pass through either station *i* or *j*, it cannot provide transfer service for i→j.

(8) $$\Delta_{ij}^{\left(2\right)}\leq 1-\Delta_{ij}^{\left(1\right)},\;i,j\in E,$$

constraint (8) ensures that if *i* and *j* are directly connected by the new bus route, no transfer is needed.

(9) $$\Delta_{ij}^{\left(2\right)}\leq \sum_{k\in S}\delta_{k}\theta_{ki},\;i,j\in E,$$

(10) $$\Delta_{ij}^{\left(2\right)}\leq \sum_{k\in S}\delta_{k}\theta_{kj},\;i,j\in E.$$

The term $\sum_{k\in S}\delta_{k}\theta_{kj}>0$ indicates that the new bus route either passes through station *i* or reaches station *j* via other bus routes. Thus, constraints (9) and (10) ensure that if the new bus route cannot reach either station *i* or *j*, no transfer is possible.

(11) $$\Delta_{ij}^{\left(2\right)}\leq \sum_{\begin{array}{c} k\in S\\ k\neq i,j \end{array}}\delta_{k}\left(\theta_{ki}+\theta_{kj}\right),\;i,j\in E,$$

Constraint (11) ensures that if there is no intermediate station on the new bus route that can reach either *i* or *j*, no transfer is possible, too, which avoids the case that the new bus route covers station *i* and passengers directly take other public transportation to reach station *j*. Subject to the constraints (1)–(11), the objective function

(12) $$\max\sum_{i,j\in E}n_{ij}\cdot \left(\Delta_{ij}^{\left(1\right)}+\frac{\Delta_{ij}^{\left(2\right)}}{\lambda }\right)$$

aims to maximize passenger flow coverage, where $n_{ij}$ is the number of passenger demands with origin station *i* and destination station *j* and λ>1 indicates that the benefit of a bus transfer is 1/λ times the benefit of a direct connection.

### 2.2. QUBO Model and CIM

The Ising model was proposed to explain phase transition phenomena in magnetic materials within statistical physics, specifically the disappearance of magnetism above a magnet’s critical temperature. However, it was later discovered to be extensible to numerous other problems in both natural and social sciences [23,26,32,33,34,35]. It remains the only model in statistical physics that simultaneously possesses three advantages: simple formulation, rich implications, and broad applicability, making it suitable for solving various combinatorial optimization problems. Generally, an Ising model can be expressed as

(13) $$H\left(\sigma \right)=-\sum_{i,j}J_{ij}\sigma_{i}\sigma_{j}-\sum_{i}h_{i}\sigma_{i},$$

where σ represents the spin variable to be determined, corresponding to a binary decision variable with possible values of −1,1, *H* denotes the Hamiltonian, *J* is the quadratic coefficient representing interaction terms, typically symmetric ($J_{ij}=J_{ji}$), and $h_{i}$ are linear coefficients representing known quantities. Mathematically, solving for the ground state energy of the Ising model constitutes an integer programming problem, which in large scale belongs to the class of NP-complete problems. This shares similarities with combinatorial optimization problems and inspires us to use quantum computers based on the Ising model to solve combinatorial optimization problems. The specific approach involves converting these problems into the Ising model framework and determining the ground state through quantum physical systems operating via spin dynamics. For a CIM, the system’s “most efficient” collective oscillation mode corresponds to the optimal solution of the given Ising problem.

In order to solve Equation (13) by the CIM we used in this study, an auxiliary spin variable $\sigma_{a}$ is introduced [32,33] and the problem is restated as

(14) $$\min_{\sigma_{a},\sigma_{1},\sigma_{2},\ldots ,\sigma_{N}}\left(-\sum_{i=1}^{N}h_{i}\sigma_{i}\sigma_{a}-\sum_{i\neq j}J_{ij}\sigma_{i}\sigma_{j}\right).$$

The linear terms are eliminated, which makes the problem suitable for CIM that does not support bias terms. The new Ising problem has two degenerate solutions:

(15) $${\left\{{\hat{\sigma }}_{i}\right\}}_{i=1}^{N},\;{\hat{\sigma }}_{a}=1\;\mathrm{and}\;{\left\{-{\hat{\sigma }}_{i}\right\}}_{i=1}^{N},\;{\hat{\sigma }}_{a}=-1,$$

where ${\left\{{\hat{\sigma }}_{i}\right\}}_{i=1}^{N}$ represents the solution to the original Ising problem. In this way, the effect of the linear bias in Equation (13) is retained in the QUBO model described below.

QUBO (Quadratic Unconstrained Binary Optimization) is one of the most widely used optimization models in quantum computing [36,37,38,39,40,41]. It unifies a rich variety of combinatorial optimization problems and can be accelerated by quantum computers to efficiently solve such problems. Moreover, the QUBO model can also represent bitwise operations, thus facilitating logic design and other operations. Usually, a QUBO model is written as

(16) $$f_{Q}\left(x\right)=\sum_{i=1}^{n}\sum_{j=1}^{i}q_{ij}x_{i}x_{j},$$

where $x_{i}\in \left\{0,1\right\}$ are binary decision variables (in QUBO problems, variables take values 0 or 1, not −1 and +1); $q_{ij}$ denotes the coefficients of the quadratic terms, representing the interactions between different variables. Solving the QUBO problem is to find the objective solution

(17) $$x^{*}=\arg\min_{x}f\left(x\right).$$

Many classic problems in theoretical computer science, such as Max-Cut, graph coloring, and partitioning, can be transformed into QUBO problems. Some machine learning models can also be embedded into the QUBO framework, including support vector machines, clustering, and probabilistic graphical models. Furthermore, due to its close connection with the Ising model, QUBO has become a core problem class in quantum annealing.

QUBO and Ising models are closely related mathematically and can be mutually transformed through appropriate variable mapping and mathematical conversion. Essentially, they describe similar binary optimization problems. The QUBO model is more convenient for modeling purposes, while the Ising model can be directly used by the CIM solver for computation. To transform between QUBO and Ising models, the following variable substitution is used

(18) $$\begin{array}{cc} \sigma_{i} & =2x_{i}-1, \end{array}$$

(19) $$\begin{array}{cc} x_{i} & =(\sigma_{i}+1)/2. \end{array}$$

QUBO models use variables with values of 0 or 1, which makes it more directly applicable to many optimization algorithms, particularly optimization methods in the field of quantum computing. To solve the BRO problem in CIM, the first step is to formulate it as a QUBO model, which can then be directly transformed into an Ising model. CIM is designed to simulate Ising model effectively and the ground energy spin configuration of the Ising model corresponds to the optimal solution. The main goal of CIM is to find this ground energy spin configuration on a sub-millisecond timescale. Because the optical CIM is designed to be all-to-all connective and programmable, the Ising model can be directly simulated and solved utilizing the controllable quantum phase transition inherent to the CIM.

In the QUBO model, each constraint is handled implicitly by adding a penalty term to the objective function. The constraint conditions Equations (1)–(11) can be transformed into the corresponding penalty terms and the original constrained classical model can be equivalently transformed into an unconstrained QUBO model [32,42]. First, the original inequality constraints are transformed into equality constraints by adding slack variables, where all slack variables are non-negative integers and in the binary form. For example, Equation (1) is first transformed to $x_{ij}+s_{ij}-a_{ij}=0.$ Next, in order to incorporate all equality constraints into the objective function so that the formulation satisfies the QUBO form, the left-hand side is squared and multiplied by a sufficiently large penalty coefficient, and this term is added to the objective function. For example, Equation (1) is transformed into $P_{1}\sum_{i,j\in E}{\left(x_{ij}+s_{ij}-a_{ij}\right)}^{2}$. In this way, when the constraints are satisfied, the corresponding terms in the objective function are zero. The objective minimization will therefore tend to drive all penalty terms to zero, meaning that all constraints are satisfied. Thus, the original model is transformed into the following QUBO model:

(20) $$H=-\sum_{i,j\in E}n_{ij}\left(\Delta_{ij}^{\left(1\right)}+\frac{\Delta_{ij}^{\left(2\right)}}{\lambda }\right)+\sum_{v=1}^{11}H_{v},$$

(21) $$H_{1}=P_{1}\sum_{i,j\in E}{\left(x_{ij}+s_{ij}-a_{ij}\right)}^{2},$$

(22) $$H_{2}=P_{2}{\left(\sum_{j\in S}x_{sj}-1\right)}^{2},$$

(23) $$H_{3}=P_{3}{\left(\sum_{i\in S}x_{it}-1\right)}^{2},$$

(24) $$H_{4}=P_{4}\sum_{i\in S\setminus \{s,t\}}{\left(\sum_{j\in S}x_{ij}-\sum_{k\in S}x_{ki}\right)}^{2},$$

(25) $$H_{5}=P_{5}{\left(\sum_{i,j\in S}d_{ij}x_{ij}+\sum_{k}2^{k}s_{1k}-L_{\max}\right)}^{2}+P_{6}{\left(\sum_{i,j\in S}d_{ij}x_{ij}-L_{\min}-\sum_{k}2^{k}s_{2k}\right)}^{2},$$

(26) $$H_{6}=P_{7}\sum_{i\in S}{\left(\delta_{i}+\sum_{m}2^{m}t_{im}-\sum_{k\in S}x_{ik}\right)}^{2},$$

(27) $$H_{7}=P_{8}\sum_{i,j\in E}{\left(\Delta_{ij}^{\left(2\right)}+r_{ij0}+2r_{ij1}-\delta_{i}-\delta_{j}\right)}^{2},$$

(28) $$H_{8}=P_{9}\sum_{i,j\in E}{\left(\Delta_{ij}^{\left(2\right)}+w_{ij}+\Delta_{ij}^{\left(1\right)}-1\right)}^{2},$$

(29) $$H_{9}=P_{10}\sum_{i,j\in E}{\left(\Delta_{ij}^{\left(2\right)}+\sum_{m}2^{m}q_{ijm}^{\left(1\right)}-\sum_{k\in S}\delta_{k}\theta_{ki}\right)}^{2},$$

(30) $$H_{10}=P_{11}\sum_{i,j\in E}{\left(\Delta_{ij}^{\left(2\right)}+\sum_{m}2^{m}q_{ijm}^{\left(2\right)}-\sum_{k\in S}\delta_{k}\theta_{kj}\right)}^{2},$$

(31) $$H_{11}=P_{12}\sum_{i,j\in E}{\left(\Delta_{ij}^{\left(2\right)}+\sum_{m}2^{m}q_{ijm}^{\left(3\right)}-\sum_{\begin{array}{c} k\in S\\ k\neq i,j \end{array}}\delta_{k}\left(\theta_{ki}+\theta_{kj}\right)\right)}^{2},$$

where $s_{ij},s_{1k},s_{2k},t_{im},r_{ij0},r_{ij1},w_{ij},q_{ijm}^{\left(1\right)},q_{ijm}^{\left(2\right)},q_{ijm}^{\left(3\right)}\in \left\{0,1\right\}$ are slack variables, and $P_{1},\ldots ,P_{12}$ are penalty parameters chosen to enforce constraints. Based on the Kaiwu SDK [43], we only need to focus on building mathematical models corresponding to their specific scenarios. The methods provided by the Kaiwu SDK can automatically determine the penalty coefficients, which will significantly reduce the difficulty of using CIM to solve practical problems.

## 3. Experiment

In this section, based on the transport data provided by Beijing Public Transport Group (Beijing, China), we investigate a real-world BRO problem, i.e., planning a new bus route in Beijing with bus transfer taken into consideration. Input data details include: passenger demand matrix $n_{ij}$ with around 40,000 non-zero OD pairs, total passenger demand of around 700,000; distance matrix $d_{ij}$ based on Euclidean distances. In the refining phase, we solve the classical model using Gurobi [44], simulated annealing (SA) [45,46,47,48,49] and Tabu search [50,51], respectively, on a classical computer (3.1 GHz Intel Core i7 CPU and 16 GB memory). Next, The BRO problem in the form of QUBO model is solved using a physical optical 100-qubits CIM manufactured by Beijing Qboson Quantum Technology Co.Ltd. In detail, the 100-qubit CIM based in Beijing operates at a wavelength of 1550 nm and has a pulse repetition rate of 100 MHz. We compare the computation performance of the CIM with that of the classical computer.

### 3.1. The Principle of CIM Solving Optimization Problems

**Figure 1:**
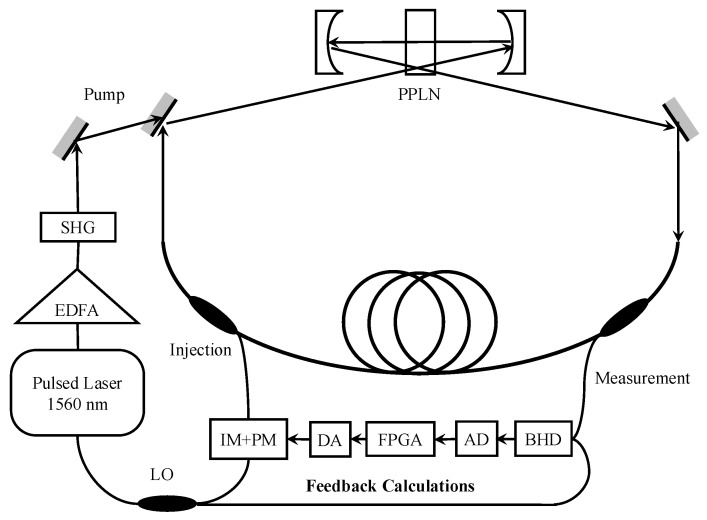
Structure and basic principle of the CIM.

The optical 100-qubits CIM used in this paper is a specialized quantum computer using a photonic dissipative architecture [18,20,21]. We show the structure and basic principle of the CIM in Figure 1. It defines the state of a qubit (|0〉 and |1〉) through the phases of optical pulses, solving the Ising model’s optimization problem via interference and oscillation of these pulses in optical parametric oscillators, utilizing the phenomenon of spontaneous symmetry breaking.

First, a pulsed laser is modulated into two pulse sequences through a coupler. One serves as the local oscillator (LO) for subsequent detection of output light phase information; the other acts as the pump pulse, serving as the system’s energy source to continuously supply energy. The optical pulses undergo second-harmonic generation (SHG), doubling the light frequency to create higher-energy light. These then pass through a periodically poled lithium niobate waveguide module (PPLN waveguide), halving the frequency again to introduce randomness in the pulse phases. Simultaneously, through degenerate optical parametric amplification (DOPA), photons accumulate and the pulses are modulated into several signal pulses with random phases.

These signals are then applied to another reference light beam using an intensity modulator (IM) and phase modulator (PM), ultimately combining with the ring cavity signals through an injection coupler for interference (constructive or destructive). This process is called degenerate optical parametric oscillation (DOPO), where IM adjusts light intensity and PM adjusts phase.

During DOPO, signal pulse phases represent spin directions in the Ising model. Initially, due to low system energy, most pulses destructively interfere and return to low-photon-number states, with phases flipping due to quantum noise. As pump pulses continuously inject energy, the system energy increases until reaching a threshold where all pulses stop destructively interfering and flipping phases, which is a quantum phenomenon called spontaneous symmetry breaking. The final phase configuration of these pulses corresponds to the Ising problem’s optimal solution. The FPGA (field-programmable gate array) reads these phases and transmits them to the host computer. For certain structured optimization problems, quantum annealing may exhibit longer time-to-solution or much lower success probability under comparable conditions  [22]. For the BRO problem, a wider range of quantum and quantum-inspired techniques, such as D-Wave’s quantum annealers [4] or Toshiba’s Simulated Bifurcation Machine [52], can be benchmarked in future studies to properly evaluate the suitability and efficiency of our solutions. The optical signals are set to propagate in the fiber loop for thousands of round trips to ensure the optical parametric oscillations reaching a steady state. For the 100-qubits CIM used for this work, the single round-trip time is 2.11 μs and the BRO problem is solved under 1000 round trips; In terms of computational energy consumption, the energy consumed per run is on the order of $24\;J=6.67\times 10^{-6}\;\mathrm{kWh}.$ For the BRO problem of the scale and complexity considered in this study, the energy consumption required by a classical computer is also negligible. However, the computational energy consumption of the CIM does not increase rapidly with problem scale or complexity, as the solving time of it is generally on the order of milliseconds [18,19]. In contrast, the solving time and energy consumption of classical computers can increase rapidly with problem scale and complexity [4]. Researchers interested in using the CIM solver can apply for scientific access through the cloud service platform [43].

### 3.2. Experimental Results

As we explained above, this real-world bus route optimization problem involves at least 60 candidate stations in Beijing with bus transfer taken into consideration. Consequently, the extremely complex and extensive public bus network of Beijing poses significant challenges for classical computers to solve the BRO problem and we choose to adopt the two-stage optimization strategy. In the pre-optimization phase, the objective function Equation (12) is solved using OR-Tools and a new bus route $R_{1}$ is designed. The new route chooses 26 stations from the 60 candidate stations. The OR-Tools output was directly used as the initial state for the refining phase. In the refining phase, we need to decide whether station *i* in $R_{1}$ is retained and Gurobi, SA, and Tabu search are used for this purpose, respectively. Specifically, the objective function in the refining phase is

(32) $$\begin{array}{c} \max\sum_{i=1}^{n}\omega_{1}\Delta t_{i}\left(1-y_{i}\right)-\omega_{2}c_{i}\left(1-y_{i}\right). \end{array}$$

If station *i* is retained, $y_{i}=1$; otherwise, $y_{i}=0$. $\omega_{1}$ is the time saving weight, whereas $\omega_{2}$ represents customer loss penalty weight. $\Delta t_{i}$ represents the total travel time reduction for all affected OD pairs after deleting station *i*, while $c_{i}$ is the number of unsatisfiable OD pairs when station *i* is removed. Thus, Equation (32) is the weighted score that measures the benefits of removing station *i* from the bus route $R_{1}$ planned in the pre-optimization phase. The solution maps showing the chosen routes in the two phases are presented in Appendix A.

**Figure 2:**
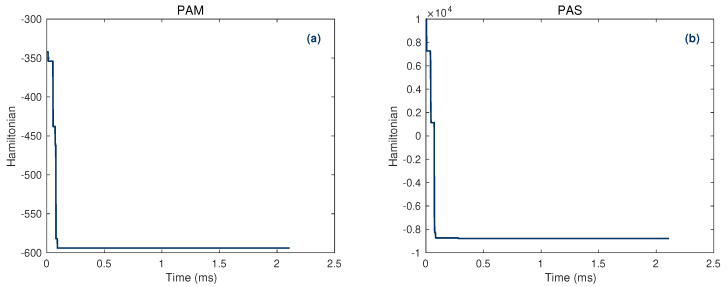
Hamiltonian time-evolution diagram for obtaining the optimal solution by CIM. (**a**) Precision reduction using the PAM method. (**b**) Precision reduction using the PAS method.

With the QUBO model established, the QUBO matrix can be generated and inputted to the CIM to solve the problem. The CIM works as a black box to output the solution, with no further manipulation needed, which makes the application of CIM very simple and promotes the practical application of quantum computing significantly. We should note that due to the hardware limitation of the 100-qubit CIM, the QUBO matrix (the input to CIM), needs to be reduced in precision. In this study, two different approaches are adopted for this purpose [53]. The precision-adaptive mutation (PAM) method is applied to the QUBO matrix *Q*, which reduces the hardware requirement by truncating floating-point elements to a lower precision, meanwhile the optimal solution of the objective function remains almost unchanged, but the degree to which the precision can be reduced to depends on inherent reducible space of the matrix *Q*. The second approach, the precision-adaptive split (PAS) method can modify the matrix to arbitrary precision, but the number of required bits of the new matrix increases rapidly with the magnitude of precision change. The Hamiltonian time-evolution diagram for obtaining the optimal solution by CIM using the two approaches are presented in Figure 2.

To quantify the acceleration capability of CIM, we define the time-saving ratio

(33) $$\tau =\frac{t_{c}-t_{\mathrm{cim}}}{t_{c}},$$

where $t_{c}$ ($t_{\mathrm{cim}}$) is the computation time of the classical computer (CIM). The computation performances of Gurobi, SA, Tabu search and CIM are listed in Table 2 and Table 3. The SA optimizer was configured with an initial temperature of 2000, a geometric cooling schedule with rate α=0.9, and a cutoff temperature of 0.1. At each temperature, 100 iterations were performed. The acceptance probability followed the standard Metropolis criterion. To allow stochastic variability across runs, no fixed random seed was used. The Tabu search optimizer was configured with a maximum of 100 iterations and all other parameters being default [43]. The computation experiments are repeated for 30 times respectively. The above configurations of SA and Tabu search are prioritized for speed and we still find they are much slower than the CIM. If the SA optimizer is configured with an initial temperature of 1000, a geometric cooling schedule with rate α=0.85, and at each temperature, 20 iterations being performed, the runtime of SA could be approximately 10 ms, but the quality of the solution (success probability) may significantly decrease. In Table 2, the PAM method is used to reduce precision. Gurobi is the fastest among the three classical solver, with an average runtime of 1.17 ms. CIM is much faster than Gurobi and the time-saving ratio is 84.06%. Both CIM and Gurobi can find the optimal solution with a 100% success probability, while the success probabilities of SA and Tabu are 53.3% and 16.7%, respectively.

In Table 3, the PAS method is used to reduce precision. SA is the fastest among the three classical solver, with an average runtime of 77.18 ms. However, SA and Tabu search can only find near-optimal solution while Gurobi can find the optimal solution with a 100% success probability. Compared to the classical solvers, the time-saving ratio of CIM is at least 97.79%. In particular, the near-optimal solution found by CIM has an extremely small optimality gap of 0.07% and CIM can find the optimal solution with 10% success probability. Furthermore, we see that for the two precision-reduction methods, the average runtime of Gurobi under the same configuration can change significantly, while the runtime of CIM remains approximately 1 ms.

## 4. Conclusions

Planning a new bus route to optimize passenger flow coverage in a global metropolis like Beijing is a challenging task. When bus transfer is taken into consideration, the existing extremely complex and extensive public bus network of Beijing leads to a problem characterized by high-dimensionality and non-linearity. As classical algorithms using classical computers struggle to deal with this kind of problem, quantum computers shed new light into this field. Utilizing the quantum phenomenon of spontaneous symmetry breaking, CIM has demonstrated its remarkable computational power in combinatorial optimization problems and capability of finding optimal solutions within milliseconds. By constructing the QUBO model of the BRO problems, we solve a complex real-world problem using CIM and demonstrate the acceleration capability of it, albeit subject to the hardware limitations of the 100-qubit CIM. Due to the search-from-below principle of CIM operation [22], we can expect that the advantage of CIM in solving large-scale optimization problems will grow rapidly along with the development of hardware capabilities of coherent Ising machine with thousands of qubits.

## Figures and Tables

**Table 1:** Mathematical notation.

*S*	Set of candidate stations for the new bus route
*E*	Set of all stations
*W*	Set of OD pairs
*A*	Set of adjacent stations
$x_{ij}$	Binary variable indicating whether the path from station *i* to station *j* is selected (*i* and *j* are adjacent stations).
$n_{ij}$	Number of passenger demands with origin station *i* and destination station *j*
$a_{ij}$	Constant indicating whether stations *i* and *j* are adjacent stations. If the distance between them is below a certain threshold, $a_{ij}=1$; otherwise, $a_{ij}=0$.
$d_{ij}$	Path length between stations *i* and *j*
$\theta_{ij}$	Constant. $\theta_{ij}=1$ indicates that station i∈S can reach target station j∈E via existing bus routes. Otherwise, $\theta_{ij}=0$. Note that $\theta_{ii}=1$.

**Table 2:** Computation performances of Gurobi, SA, Tabu search, and CIM with the PAM method.

PAM	CIM	Gurobi	SA	Tabu
Average Runtime ± SD (ms)	0.19 ± 0.16	1.17 ± 0.32	69.57 ± 6.26	73.86 ± 8.01
Time-saving ratio (%)		84.06	99.73	99.75
Success Probability (%)	100	100	53.3	16.7

**Table 3:** Computation performances of Gurobi, SA, Tabu search and CIM with the PAS method.

PAS	CIM	Gurobi	SA	Tabu
Average Runtime ± SD (ms)	1.71 ± 1.50	999.87 ± 14.67	77.18 ± 8.89	121.42 ± 9.08
Time-saving ratio (%)		99.83	97.79	98.60
Optimality Gap (%)	0.07	0	1.63	0.70
Success Probability (%)	10	100	0	0

**Figure A1:**
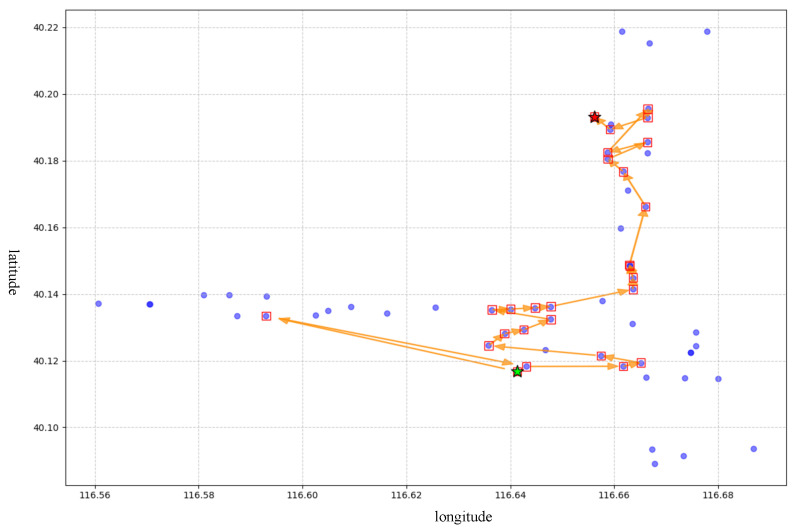
The solution map showing the chosen route $R_{1}$ in the pre-optimization phase. The blue dots represent the candidate stations. The chosen route in the pre-optimization phase consists of 26 stations. The blue circles are the candidate stations and the green (red) star represents the start (terminal) station.

**Figure A2:**
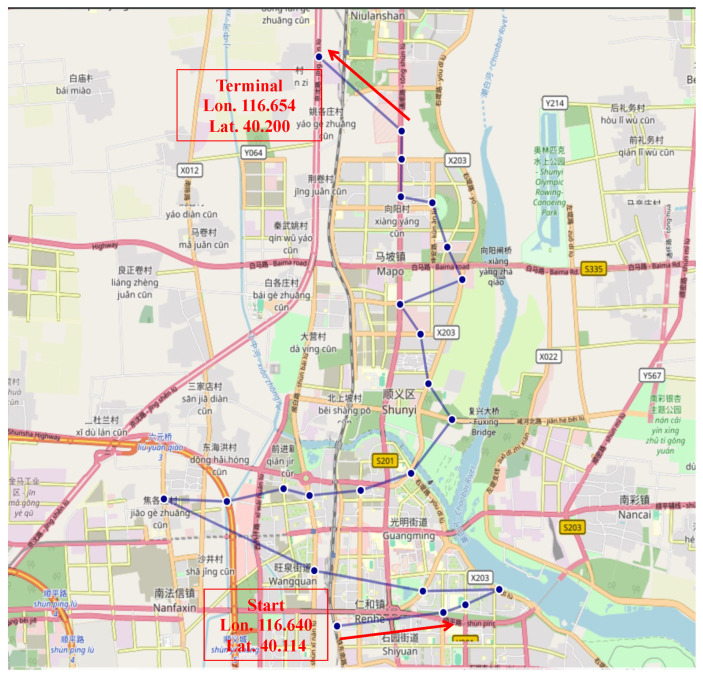
The solution map showing the chosen route in the refining phase using the PAM method to reduce precision.

**Figure A3:**
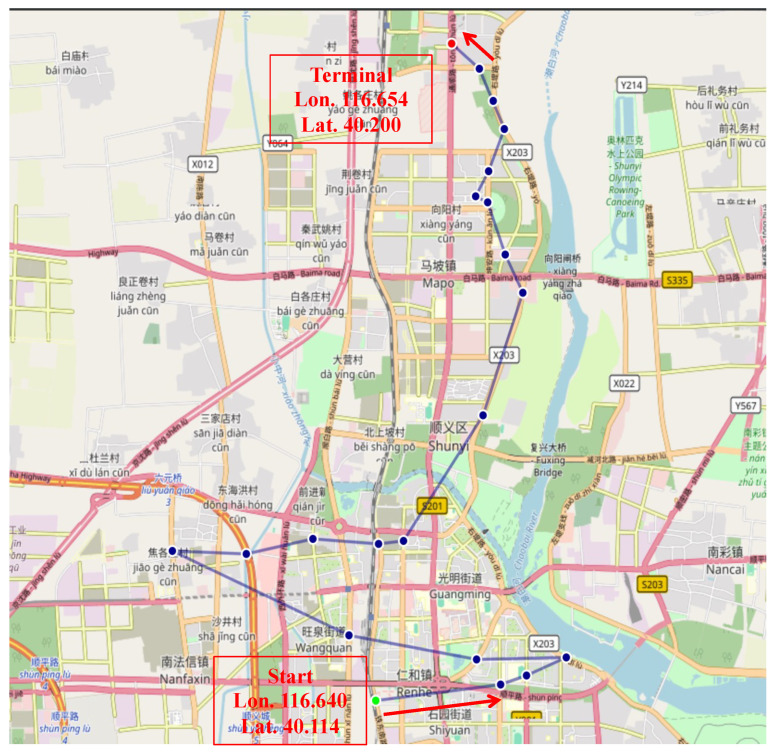
The solution map showing the chosen route in the refining phase using the PAS method to reduce precision.

## Data Availability

The data and Supplementary Materials presented in this study are available on request from the corresponding author.

## Supplementary Materials

The following supporting information can be downloaded at: https://www.mdpi.com/article/10.3390/e27090953/s1, Table S1: Experimental results.

## Abbreviations

    The following abbreviations are used in this manuscript:

CIM	Coherent Ising Machine
BRO	Bus Route Optimization
QUBO	Quadratic Unconstrained Binary Optimization
TND	Transport Network Design
SA	Simulated Annealing

## Appendix A. The Chosen Routes and Pseudocode

The solution maps with OpenStreetMap [54] showing the chosen routes in the two phases are presented below.

To improve clarity, we present the pseudocode to illustrate the two-stage pipeline OR-Tools + Gurobi, Simulated Annealing or Tabu search.

**Algorithm A1** Two-Stage Pipeline: OR-Tools + Gurobi, Simulated Annealing or Tabu search
1: **Stage 1: OR-Tools pre-optimisation** 2: Input: station sets … 3: Model ← OR-Tools 4: Define binary variables 5: Add constraints 6: Solver ← CP-SAT solver() 7: Set solver parameters (default configuration) 8: Solution_or_tools ← solver … 9: **Stage 2: Refinement** 10: Use encoded solution from previous stage: Solution_or_tools 11: Gurobi, Simulated Annealing or Tabu search 12: Set parameters 13: Iterations and selection of solutions … 14: Output
